# Health-Promoting Lifestyle and Associated Factors among Medical Sciences Students in Kermanshah, Iran: A Cross-Sectional Study

**DOI:** 10.1155/2021/6691593

**Published:** 2021-04-26

**Authors:** Baharak Azami Gilan, Maryam Janatolmakan, Hossein Ashtarian, Mansour Rezaei, Alireza Khatony

**Affiliations:** ^1^Student Research Committee, Kermanshah University of Medical Sciences, Kermanshah, Iran; ^2^Clinical Research Development Center, Imam Reza Hospital, Kermanshah University of Medical Sciences, Kermanshah, Iran; ^3^Health Education Department, Kermanshah University of Medical Sciences, Kermanshah, Iran; ^4^Social Development and Health Promotion Research Center, Health Institute, Kermanshah University of Medical Sciences, Kermanshah, Iran; ^5^Infectious Diseases Research Center, Kermanshah University of Medical Sciences, Kermanshah, Iran

## Abstract

**Background:**

Lifestyle includes routine and daily living activities affecting an individual's health. The present study aimed at evaluating the health-promoting lifestyle profile (HPLP) of medical sciences students of Kermanshah, Iran.

**Methods:**

In this cross-sectional study, 343 medical sciences students were enrolled by the stratified random sampling method. The data collection tools were demographic information form and the HPLP-II questionnaire. Data were analyzed using descriptive and analytical statistics.

**Results:**

The mean overall HPLP-II score of the subjects was 2.25 ± 0.44 out of 4. Of the six HPLP-II dimensions, the highest and lowest scores belonged to interpersonal relations and physical activity, respectively. The mean overall HPLP-II score was statistically different in terms of gender, marital status, smoking habits, and economic status (*P* ≤ 0.05).

**Conclusion:**

HPLP-II level was moderate in most of the students, and health-promoting behaviors, in the physical activity dimension, were in a low state. The results emphasized the need for interventions to improve students' lifestyles.

## 1. Introduction

Lifestyle includes daily routines that become habitual and affect physical and mental health [1]. Measures such as a healthy diet, adequate sleep and activity, weight control, and lack of smoking and alcohol consumption constitute lifestyle [2, 3]. An unhealthy lifestyle is one of the main risk factors for chronic diseases and premature death [4, 5]. According to the US Department of Health and Human Services, unhealthy behavior and lifestyle is one of the leading causes of death [6]. Improvement of lifestyle leads to satisfaction, success at work, and physical health, called the health-promoting lifestyle profile (HPLP). It consists of six dimensions as follows: physical activity, nutrition, health responsibility, spiritual growth, interpersonal relations, and stress management [2, 7, 8]. Students often neglect to maintain a healthy lifestyle, which puts them at risk of old age diseases [9]. Likewise, an unhealthy diet and low physical activity are reported as one of the dimensions of an unhealthy lifestyle in students [10–12]. Excessive consumption of fast foods, low physical activity, an irregular sleep pattern, and smoking are among the unhealthy behaviors that negatively affect students' health [13, 14]. In 2016, a study in the United States showed that 40% of students were overweight, and 19% had high serum cholesterol levels due to an unhealthy lifestyle [15]. Likewise, a study in the UK (2017) reported that a small number of students followed five healthy lifestyle behaviors, including a healthy diet, regular physical activity, maintaining a healthy weight, and not smoking [16]. Therefore, it is essential to observe HPLP to prevent chronic diseases [14, 17]. Concerning the importance of the issue and the lack of information about the HPLP of students of Kermanshah University of Medical Sciences (KUMS), the current study was designed. In this study, we sought to answer the following questions: what are the mean scores of HPLP-II and its dimensions and what is the relationship between the mean HPLP-II and the personal characteristics of students?

## 2. Methods

### 2.1. Study Design

This descriptive-analytic cross-sectional study was conducted in six faculties of Kermanshah University of Medical Sciences. The study was performed according to STROBE instructions.

### 2.2. Study Questions

In this study, we sought to answer the following questions:

1. What are the mean scores of HPLP-II and its dimensions?
2. What is the relationship between the mean HPLP-II and the personal characteristics of students?

### 2.3. Sample and Sampling Method

The study population included all the students studying in the affiliated faculties of Kermanshah University of Medical Sciences (*n* = 3725), including medicine, nursing and midwifery, health, paramedical, pharmacy, and dentistry. The sample size was calculated based on the results of the study of Rezaei-Adaryani and Rezaei-Adaryani [18], and using the formula *n* = ((*σ*)^2^*∗Z*_(*α*/2)_^2^)/*d*^2^ equal to 312 people (*d* = 0.04, *σ*=0.36,  *α*=1.96). To cover the missing data, 31 people (10%) were added to the sample size (343 people in total). The sample size was proportional to the total number of students in each faculty. The number of students in each faculty was as follows: medicine, 95 people; nursing and midwifery, 52 people; paramedical, 88 people; health, 57 people; dentistry, 18 people; pharmacy, 33 people.

Inclusion criteria were as follows: willingness to participate in the study and lack of lifestyle-affecting diseases (i.e., diabetes, multiple sclerosis, and musculoskeletal disorders). The stratified random sampling method was used to select the subjects, and each faculty formed a stratum. The simple random sampling method using a table of random numbers was employed to select the subjects in each stratum.

### 2.4. Study Instruments

The demographic information form and HPLP-II questionnaire were used as data collection instruments. The demographic information form included nine items on age, gender, field of study, place of residence, marital status, economic status, weight, height, and smoking habits. The HPLP-II questionnaire was used to evaluate HPLP. Studies by Savarese et al. and Petrash & Murtazina evaluated the internal consistency of the questionnaire and reported its Cronbach's alpha as 0.94 and 0.88, respectively [7, 19]. The Persian version of HPLP-II was psychometrically assessed in studies by Soleimani Moghadam et al. and Tanjani et al., reporting Cronbach alpha of 0.80 and 0.78, respectively [20, 21].

The HPLP-II includes 52 items scored based on a four-point Likert scale as never [1], sometimes [2], most often [3], and regularly [4]. It measures HPLP-II in six dimensions of nutrition (nine items), physical activity (eight items), health responsibility (nine items), stress management (eight items), interpersonal relations (nine items), and self-realization (nine items). The mean score earned by each person was calculated out of four and a mean score of ≥2.5 was considered a positive response [18]. The economic status of the family was categorized in the present study based on the monthly income as high (≥$ 201), middle ($101–$ 200), and low (≤$100).

### 2.5. Data Collection Method

After obtaining approval from the Ethics Committee of KUMS, the researcher referred to the Education Department of the affiliated faculties to enroll the eligible students. Accordingly, the study objectives were explained to students, and their consent for participation in the study was obtained. Then, the questionnaires were distributed among the subjects and collected after completion.

### 2.6. Statistical Analysis

Data were analyzed using SPSS software version 16. Descriptive statistics (mean, standard deviation, and frequency tables) and analytical statistics (Mann–Whitney U test, Kruskal–Wallis H test, and Kolmogorov–Smirnov test) were used to analyze the data. Kolmogorov–Smirnov test was used to evaluate the normality of the HPLP-II variable and its subset, and the results showed an abnormal distribution of these variables.

The Mann–Whitney *U* test was utilized to compare the mean HPLP-II scores in terms of the two-state qualitative variables, including gender, marital status, place of residence, and smoking habits. The Kruskal–Wallis H test was used to compare the mean HPLP-II scores in terms of the multistate qualitative variables, including the field of study, BMI, and family economic status. The level of significance for all tests was less than 0.05.

### 2.7. Ethical Consideration

The ethics committee of Kermanshah University of Medical Sciences approved the study with the code KUMS.REC.1399.167 (approval date 2020-05-11). Written and informed consent was obtained from all participants. Emphasis was placed on the confidentiality of participants' information.

## 3. Results

In the current study, the response rate was 100%. According to the results, 173 subjects (50.4%) were females, 323 (94.2%) single, 306 (89.2%) dormitory residents, and 95 (27.7%) medical students, and 218 (63.6%) had a medium family income (Table 1). The mean overall HPLP-II score was 2.25 out of 4. Among the six HPLP-II dimensions, the highest and lowest mean scores belonged to interpersonal relations (2.60 ± 0.52) and physical activity (1.97 ± 0.62), respectively (Table 2). The results showed that the mean HPLP-II score of the married subjects was significantly higher than that of single ones (2.47 ± 0.38 vs. 2.24 ± 0.43), and the difference was statistically significant. In the present study, the mean HPLP score of male subjects was significantly higher than that of females (2.34 ± 0.40 vs. 2.16 ± 0.45). Students who lived with their families had a higher mean HPLP-II score than those living in dormitories (2.23 ± 0.43 vs. 2.40 ± 0.47), and the difference was statistically insignificant. The results showed that the mean HPLP-II score of nonsmoking students was significantly higher than that of smoking ones (2.30 ± 0.43 vs. 2.00 ± 0.42). In the present study, subjects with high economic status (≥$ 201) had a higher mean HPLP-II score than those with middle or low status ($ 101–$ 200, and ≤$ 100, respectively). No significant relationship was found between the mean HPLP-II score and age and BMI variables (Table 3).

## 4. Discussion

According to the results, the mean overall HPLP-II score was at a moderate level, in line with the results of studies by Mehri et al. and Montazeri et al. in Iran, Al-Qahtani et al. in Saudi Arabia, and Al‐Kandari in Kuwait [2, 22–24]. However, in studies by Pakseresht et al. in Iran and Al-Qahtani in Saudi Arabia, the majority of the students had a poor HPLP-II [1, 25]. However, in studies by Lolokote et al. in China and Borle et al. in India, HPLP was reported as good [26, 27]. These results emphasize the need for planning to train health-promoting behaviors in students.

The results showed that married subjects had a higher mean HPLP-II score than single ones. The finding was consistent with that of Lolokote et al. in China and inconsistent with that of Mehri et al. in Iran [2, 26]. It indicates that marital status can influence the promotion of health.

In the present study, the mean overall HPLP-II score of the male subjects was significantly higher than that of females, consistent with the results of studies by Mehri et al. in Iran, Almutairi et al., Dhiman and Chawla, and Alzahrani et al. in Saudi Arabia and Paudel et al. in Nepal [2, 12, 14, 28, 29]. Given that the HPLP-II has six domains, males and females can gain different scores in each due to physical, mental, and psychological differences.

The results showed that students who lived with their families had a higher mean overall HPLP-II score than the ones living in dormitories. This result was consistent with that of Mehri et al. in Iran and inconsistent with that of Alzahrani et al. in Saudi Arabia [2, 12]. Students who live in dormitories benefit from fewer facilities and less family support compared to the ones living with their families, which can affect their HPLP-II scores.

In the current study, the mean overall HPLP-II score of nonsmoking students was significantly higher than that of smoking ones, consistent with the results of Alzahrani et al. in Saudi Arabia, Lolokote et al. in China, and Aynaci and Akdemir in Turkey [12, 26, 30]. Smoking is an unhealthy behavior with negative impacts on lifestyle.

In the present study, students with a high economic level had a significantly higher mean HPLP-II score than the ones with middle to low levels. These results are in line with studies by Pakseresht et al. in Iran, Alzahrani et al. in Saudi Arabia, Lolokote et al. in China, and Dhiman and Chawla in Saudi Arabia [1, 12, 26, 28]. Financial concerns significantly affect HPLP and, therefore, financial support of students from both the family and university seems essential.

In the present study, the mean HPLP-II score was at a moderate level in all the age groups, and no significant difference was observed among them. This finding was consistent with the result of Alzahrani et al. in Saudi Arabia [12] and inconsistent with those of Al-Qahtani and Al-Qahtani in Saudi Arabia, Lolokote et al. in China, Borle et al. in India, and Kurnat-Thoma et al. in the United States [24–27, 31]. Therefore, it is necessary to plan health promotion activities for all age groups of students.

The results showed that the mean overall HPLP-II score had no significant differences among BMI groups (underweight, normal, overweight, and obese), consistent with the results of Alzahrani et al. in Saudi Arabia [12] and inconsistent with those of Mehri et al. in Iran, Lolokote et al. in China, and Çakaroğlu et al. in Turkey [2, 26, 32]. Although no significant relationship was found in the present study between the mean HPLP-II score and BMI, it can be used as an index of HPLP-II.

In the present study, the mean HPLP-II score was at a moderate level in students of all KUMS faculties, and no significant difference was found among them. In the study by Pakseresht et al. in Iran, among medicine, dentistry, health, nursing, and paramedical faculties, the paramedical faculty had the highest mean score in interpersonal relations and spiritual growth dimensions [1]. The study by Almutairi et al. in Saudi Arabia showed a significant difference between the schools of medicine and sciences in terms of health responsibility [14]. The authors of the present study believe that medical students are role models and should have good HPLP-II, considering the nature of their chosen profession.

In the present study, among the six HPLP-II domains, the highest and lowest scores belonged to interpersonal relations and physical activity, respectively, consistent with the study by Mak et al. in China [33], although some studies reported that the highest mean scores are related to interpersonal relations [2, 29, 34] and spiritual growth [12, 28, 29, 34], and the lowest to physical activity [2, 12, 29, 34], nutrition [2], and health responsibility dimensions [28, 29]. Different results may be due to cultural and educational differences among universities. These differences highlight the importance of conducting further research and applying the results to plan macro-educational and health policies.

This study faced several limitations. One limitation was the possibility of social desirability bias. In this type of bias, instead of giving correct answers to questions, respondents give answers that are more acceptable to others and society. Considering the possibility of social desirability bias, the questionnaires were anonymous to minimize the impact. The main limitation of the cross-sectional study is that the temporal relationship between the exposure and outcome variables cannot be determined because the exposure and outcome variables are measured simultaneously. Therefore, due to the nature of cross-sectional studies, it is not possible to determine the causal relationship between the variables of this study. Considering the effect of personal and cultural variables on HPLP-II, caution should be exercised in generalizing the results.

## 5. Conclusion

Most students had moderate HPLP-II. The highest and lowest scores of HPLP-II dimensions belonged to interpersonal relations and physical activity, respectively. There was a significant relationship between the mean HPLP-II score and the variables of gender, marital status, economic status, and smoking habits; however, it was insignificant in terms of BMI and age groups. It is suggested to evaluate the effect of interventions on students' HPLP-II in future studies.

## Figures and Tables

**Table 1:** Demographic characteristics of students (*n* = 343).

Variables		*N* (%)
Sex	Female	173 (50.4)
	Male	170 (49.6)

Marital status	Single	323 (94.2)
	Married	20 (5.8)

Age (years)	18–22	205 (59.8)
	23–27	120 (35.0)
	≥28	18 (5.2)

Residence	Dormitory	306 (89.2)
	House	37 (10.8)

Body mass index	Weight loss (≤18.4)	24 (7.0)
	Normal (18.5–24.9)	278 (81.0)
	Overweight (25–29.9)	37 (10.8)
	Obese (≥30)	4 (1.2)

Monthly income, in dollar	Low (≥100)	34 (9.9)
	Medium (101–200)	218 (63.6)
	High (≥201)	91 (26.5)

School	Paramedical	88 (25.6)
	Health	57 (16.6)
	Nursing and midwifery	52 (15.2)
	Dentistry	18 (5.3)
	Medical	95 (27.7)
	Pharmacy	33 (9.6)

Smoking	Yes	41 (12.0)
	No	302 (88.0)

**Table 2:** Distribution of health-promoting lifestyle profile II (HPLP-II).

Variables		Mean ± SD^a^	Min^b^-Max^c^	Q_3_^d^-Q_1_^e^
Health-promoting lifestyle subsets	Health responsibility	2.10 ± 0.54	1.00; 3.67	2.44; 1.66
	Physical activity	1.97 ± 0.62	1.00; 4.00	2.37; 1.50
	Nutrition	2.16 ± 0.50	1.00; 3.67	2.44; 1.77
	Self-realization	2.54 ± 0.63	1.00; 4.00	3.00; 2.11
	Interpersonal relationship	2.60 ± 0.52	4.00; 1.37	2.88; 2.22
	Stress management	2.10 ± 0.46	1.00; 3.63	2.37; 1.75
	Overall lifestyle points	2.25 ± 0.44	1.25; 3.64	2.53; 1.92

Note: ^a^standard deviation; ^b^minimum; ^c^maximum; ^d^first quarter; ^e^third quarter.

**Table 3:** Comparison of health-promoting lifestyle profile II (HPLP-II) among students in terms of demographic characteristics.

Variables		Mean ± SD^a^	CI 95%^b^	Test results
Sex	Female	2.16 ± 0.45	2.09–2.23	*Z* = -3.96^c^ *P*=.0001
	Male	2.34 ± 0.40	2.28–2.40	

Age (years)	18–22	2.28 ± 0.43	2.22–2.37	*X* ^2^ = 1.63 *P*=.441
	23–27	2.21 ± 0.44	2.13–2.29	
	≥28	2.20 ± 0.52	1.86–2.01	

Marital status	Single	2.24 ± 0.43	2.19–2.29	*Z* = 2.24 *P*=.025
	Married	2.47 ± 0.38	2.28–2.64	

Residence	Dormitory	2.23 ± 0.43	2.18–2.28	*Z* = 1.91 *P*=.063
	House	2.40 ± 0.47	2.40–2.56	

Smoking	Yes	2.00 ± 0.42	1.86–2.13	*Z* = 4.00 *P* ≤ .0001
	No	2.30 ± 0.43	2.24–2.33	

Body mass index	Weight loss (≤18.4)	2.22 ± 0.44	2.04–2.41	*X* ^2^ = 3.33^d^ *P*=.343
	Normal (18.5–24.9)	2.24 ± 0.42	2.19–2.29	
	Overweight (25–29.9)	2.30 ± 0.49	2.13–2.46	
	Obese (≥30)	2.70 ± 0.50	1.86–3.47	

Monthly income, in dollar	Low (≥100)	2.07 ± 0.40	1.93–2.21	*X* ^2^ = 737.28 *P*=.0001
	Medium (101–200)	2.19 ± 0.39	2.14–2.24	
	High (≥201)	2.43 ± 0.47	2.26–2.59	

School	Paramedical	2.25 ± 0.38	2.02–2.49	*X* ^2^ = 24.37 *P*=.082
	Health	2.29 ± 0.47	1.69–3.00	
	Nursing and midwifery	2.20 ± 0.47	2.00–2.41	
	Dentistry	2.51 ± 0.33	2.35–2.68	
	Medical	2.19 ± 0.45	1.92–2.46	
	Pharmacy	2.13 ± 0.39	1.99–2.27	

Note: ^a^standard deviation; ^b^confidence interval; ^c^based on Mann–Whitney *U* test; ^d^based on Kruskal–Wallis H test.

## Data Availability

The identified datasets analyzed during the current study are available from the corresponding author upon reasonable request.
